# Therapeutic effects and mechanisms of neutrophil membrane-coated MXene (Ti_**3**_C_**2**_T_**x**_) nanoparticles for sepsis treatment

**DOI:** 10.1080/10717544.2026.2752680

**Published:** 2026-11-04

**Authors:** Yu Chen, Wenjing Wu, Siyu Xia, Chao Wang, Jingjing Li

**Affiliations:** a Department of General Practice, Affiliated Hospital of Nantong University, Medical School of Nantong University, Nantong, China

**Keywords:** Sepsis, Ti_3_C_2_T_x_, neutrophil membrane, nanodrug, multimechanistic synergy

## Abstract

Sepsis, a life-threatening organ dysfunction from a dysregulated infection response, remains a high-mortality global health burden characterized by the systemic overproduction of pro-inflammatory mediators and reactive oxygen and nitrogen species. Timely anti-infective/anti-inflammatory interventions in the early onset window drastically improve sepsis prognosis, yet current therapeutic efficacy remains severely constrained by the global antibiotic resistance crisis. As a two-dimensional nanomaterial, Ti_3_C_2_T_x_ MXene displays potent intrinsic antimicrobial and anti-inflammatory activities. We herein engineer a neutrophil membrane-coated Ti_3_C_2_T_x_ nanoplatform (NM@Ti_3_C_2_T_x_ NPs) for targeted sepsis therapy. Experimental validation confirms this formulation mitigates sepsis-triggered oxidative stress injury via synergistic antioxidant pathways, with favorable colloidal stability and confirmed in vitro biocompatibility. In CLP-induced murine sepsis models, it attenuates systemic pro-inflammatory cascades, alleviates hypothermia and multi-organ damage, and improves survival. Collectively, this multifunctional platform integrating targeted antibacterial action and oxidative stress modulation presents a promising candidate for sepsis translational therapy.

## 1. Introduction

As defined by the Sepsis 3.0 international consensus, sepsis is a life-threatening organ dysfunction driven by pathogen-induced dysregulation of host inflammatory and immune pathways, with bacterial infection as its primary etiology. It globally affects approximately 49 million people annually, with ICU mortality reaching 25%–50% due to uncontrolled systemic hyperinflammation, immune suppression, and subsequent multi-organ failure (Rudd et al. 2020). Even with standard supportive regimens covering antibiotics, vasopressors, and fluid resuscitation, current sepsis therapies cannot resolve its dual pathological drivers: the initial pro-inflammatory cytokine storm (TNF-*α*, IL-1β, and IL-6) and subsequent immunosuppression featuring neutrophil dysfunction and lymphocyte apoptosis (Liu et al. 2022; Gao et al. 2025a). Strikingly, 40% of sepsis survivors face 3-month rehospitalization driven by residual inflammatory mediators that trigger recurrent cytokine storms. These unmet clinical demands highlight an urgent need for multi-target therapies that simultaneously eliminate pathogens, suppress hyperinflammation, and restore immune homeostasis (Shankar-Hari et al. 2020).

To engineer targeted, effective anti-sepsis regimens, the core cellular regulators governing sepsis onset and progression must first be precisely defined. Among all immune cell populations involved in sepsis pathogenesis, macrophages act as the central orchestrators, bridging pathogen invasion, inflammatory bursts, and downstream organ injury (Qiu et al. 2019a). In early-stage sepsis, pathogen- and damage-associated molecular patterns (PAMPs, including LPS, DAMPs, including mtDNA) activate macrophage Toll-like receptors (TLRs), initiating NF-κB signaling cascades and the downstreampro-inflammatory cytokine storm (Muendlein et al. 2021; Aydin et al. 2022; Yan et al. 2023). Concurrently, excessive reactive oxygen species (ROS) generated via oxidative stress exacerbate cellular damage (Feng et al. 2021), activate the NLRP3 inflammasome, and amplify inflammatory cascades (Danielski et al. 2020; Wu et al. 2021). Paradoxically, sustained inflammation in sepsis induces macrophage immune paralysis —a well-established model superseding the outdated simple M2 polarization framework — marked by blunted pro-inflammatory responses, impaired bacterial clearance, and aberrant upregulation of immunosuppressive mediators, including IL-10 (Chen et al. 2021). This dysfunctional state impairs pathogen clearance and tissue repair, highlighting macrophages’ dual role as both inflammatory initiators and functionally compromised therapeutic targets in sepsis pathology. Targeted macrophage reprogramming coupled with ROS scavenging has accordingly emerged as a core anti-sepsis therapeutic avenue (Marshall 2014; Cui et al. 2023; Sun et al. 2024; Peng et al. 2025).

This dual-target strategy has a solid therapeutic rationale, yet conventional implementations face unresolvable translational bottlenecks: poor inflammatory lesion targeting, insufficient local accumulation, and high off-target toxicity. Recent nanotechnology advances provide a viable path to break these clinical barriers (Fink and Warren 2014; Koide et al. 2021). Cell membrane-coated NPs, particularly NM@Ti_3_C_2_T_x_ NPs (Gao et al. 2017; Chu et al. 2018; Zhao et al. 2022), inherit innate immune cell biomimetic properties (Wang et al. 2018; Zhang et al. 2024). Via surface integrin (CD11b/CD18)-ICAM-1 interactions on the inflamed endothelium, these particles actively target infectious foci, boost local drug accumulation, and reduce off-target toxicity (Korin et al. 2012; Zhang et al. 2018; Cheng et al. 2021; Ma et al. 2022; Ma et al. 2024), with documented approximately 5-fold higher antibiotic delivery efficiency in sepsis models (Kim and Sahay 2022; Zhang et al. 2022). This superior performance originates from the fully preserved native functional components of the coated neutrophil membrane, which retains the complete structural and functional signature of intact neutrophil plasma membrane. The neutrophil coating on NM@Ti_3_C_2_T_x_ NPs fully preserves the intact structure and core functional components of native neutrophil plasma membrane, featuring an inherent asymmetric phospholipid bilayer framework: the outer leaflet is dominated by phosphatidylcholine and sphingomyelin, while the inner leaflet primarily consists of phosphatidylserine and phosphatidylethanolamine. The retention of this native asymmetric distribution not only safeguards membrane structural integrity but also provides a physiological native microenvironment to support the correct folding of surface functional proteins. The retained native neutrophil surface proteins embedded in the coating form the molecular foundation for the NPs’ three core capabilities: inflammatory lesion targeting, pathogen binding, and immune evasion. Chemotaxis and adhesion molecules on the membrane sense local infection-site chemokine gradients to mediate active enrichment in inflamed tissue; pathogen recognition proteins boost NP-pathogen affinity, amplifying local antibacterial performance; and immune evasion proteins inhibit the non-specific mononuclear phagocyte system clearance, extending blood circulation time and improving overall biocompatibility. In this work, neutrophil plasma membranes were gently isolated via low-temperature hypotonic lysis and sequential gradient centrifugation. The strict cryogenic workflow prevents membrane protein denaturation, fully preserving all core functional components for subsequent coating on Ti_3_C_2_T_x_ NPs. The resulting intact core-shell architecture integrates native neutrophil membrane biofunctions and intrinsic Ti_3_C_2_T_x_ nanomaterial activities (Oroojalian et al. 2021). With the neutrophil membrane shell design established, we next characterize the Ti_3_C_2_T_x_ core’s intrinsic therapeutic properties that underpin the overall anti-sepsis efficacy. As a typical 2D transition metal carbide MXene, Ti_3_C_2_T_x_ exhibits prominent antibacterial and anti-inflammatory activities, making it a highly promising anti-sepsis candidate (Rasool et al. 2016; Zheng et al. 2021). Its antibacterial effect mainly originates from sharp-edge mediated physical destruction of bacterial membranes. Meanwhile, its surface functional groups (-OH, -F) exert anti-inflammatory activity by neutralizing lipopolysaccharide (LPS) endotoxin and blocking the downstream TLR4/NF-κB signaling pathway (Zheng et al. 2020; Huang et al. 2022; Wu et al. 2023; Iravani and Varma 2023). Ti_3_C_2_T_x_ nanozymes further augment therapeutic performance via ROS scavenging and proinflammatory cytokine suppression, proven to improve survival in preclinical sepsis models (Ye et al. 2022; Liu et al. 2024). Its native biosafety is guaranteed by biocompatible titanium and endogenous physiological carbon. LiF/HCl in-situ etched d-Ti_3_C_2_T_x_ features a low-defect, large-lateral-size microstructure, which reduces residual fluorine terminations and associated biological risks. The product exhibits negligible cytotoxicity even at elevated concentrations, setting a high biosafety benchmark for MXene-based therapeutic nanomaterials.

Nevertheless, current strategies still face limitations in biocompatibility, targeting precision, and single-mechanism-induced immune suppression. We hereby engineer NM@Ti_3_C_2_T_x_ NPs for synergistic anti-sepsis infection and inflammation modulation. This biomimetic nanoplatform encompasses three core functions: (1) active targeting: native neutrophil membrane proteins specifically bind inflammatory endothelial molecules to enable targeted lesion accumulation and boost local therapeutic delivery; (2) synergistic anti-infective/anti-inflammatory effects: endogenous neutrophil antimicrobial peptides exert independent bactericidal activity, while the Ti_3_C_2_T_x_ core physically disrupts pathogens, neutralizes LPS, scavenges excess ROS, and suppresses TLR4/NF-κB-driven cytokine storm; and (3) immune reprogramming: co-loaded immunomodulators promote macrophage polarization toward the pro-repair M2 phenotype, accelerating inflammation resolution. Different from conventional single-target strategies, this dual-bioinspired NM@Ti_3_C_2_T_x_ design integrates the neutrophil membrane’s inherent inflammation tropism and Ti_3_C_2_T_x_’s multifunctional bioactivity to coordinately regulate antibacterial, antioxidant, and immunoregulatory processes. This multi-target design precisely matches the complex multifactorial pathophysiology of sepsis, forming a promising therapeutic paradigm for subsequent exploration (Figure 1).

**Figure 1.**
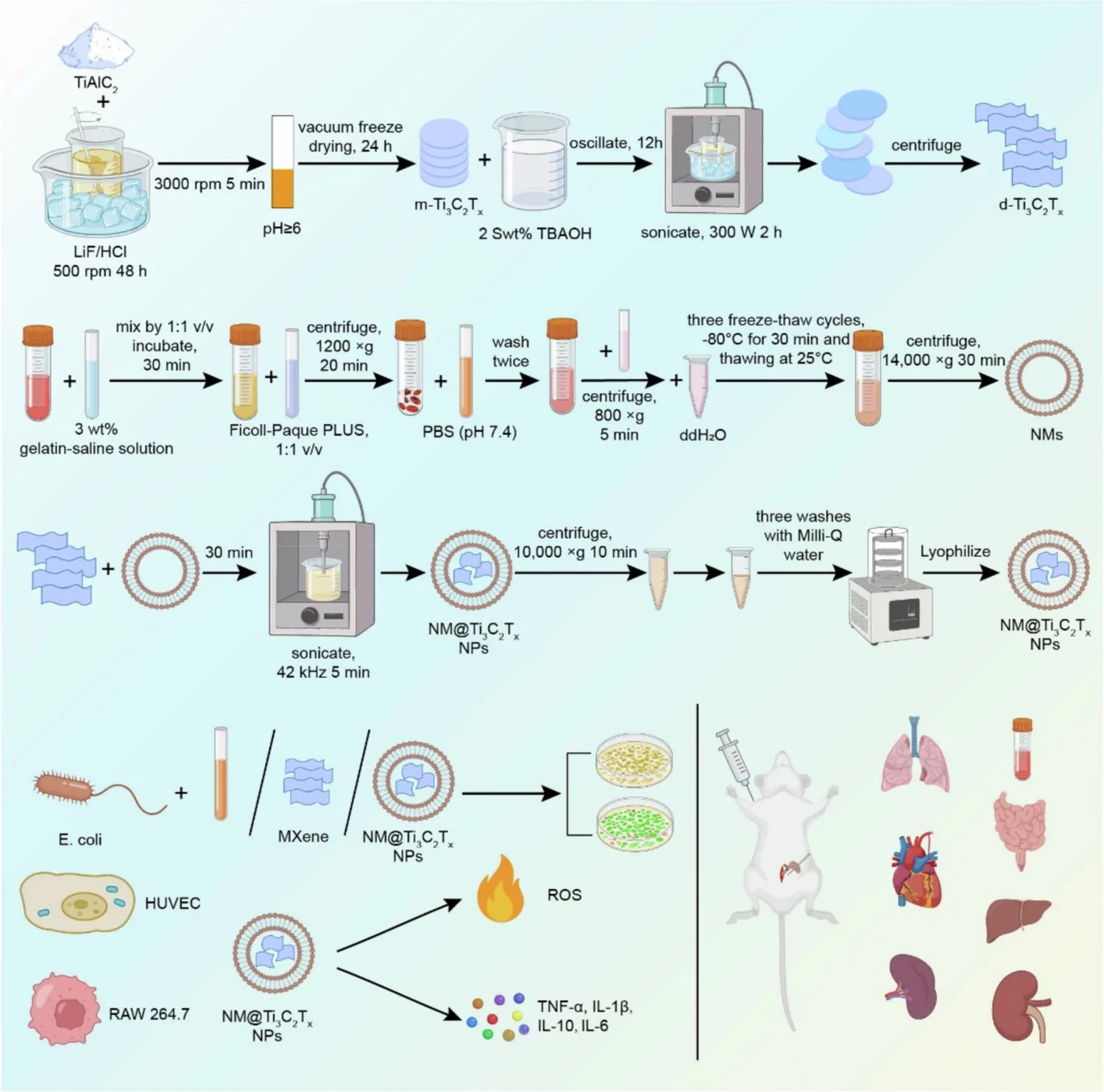
Schematic illustration of NM@Ti_3_C_2_T_x_ NPs synthesis and validation of their antibacterial and anti-inflammatory capabilities in vitro and in vivo.

## 2. Materials and methods

### 2.1. Experimental procedures and methods

#### 2.1.1. Synthesis of d-Ti_3_C_2_T_x_

One gram of Ti_3_AlC_2_ powder was slowly added to a preformulated etching solution of 3.2 g LiF dissolved in 40 ml 9 M HCl, then stirred at 500 rpm under ice cooling for 48 h. After the reaction completion, the dispersion was repeatedly centrifuged with deionized water at 3000 rpm for 5 min until the supernatant pH ≥ 6, and the precipitate was vacuum freeze-dried for 24 h to harvest multilayer m-Ti_3_C_2_T_x_. For delamination, the product was dispersed in 50 ml 25 wt% tetrabutylammonium hydroxide (TBAOH) and ultrasonicated at 300 W for 2 h in an ice bath. After removing unreacted residues via centrifugation, the single-layer d-Ti_3_C_2_T_x_ dispersion was obtained, whose single-layer nature was confirmed via the Tyndall effect.

#### 2.1.2. Isolation of neutrophil membranes (NMs)

Fresh EDTA-anticoagulated mouse blood was mixed 1:1 with 3 wt% gelatin-saline, incubated for 30 min to precipitate erythrocytes. The supernatant was overlaid onto equal-volume Ficoll-Paque PLUS, centrifuged at 1200 × g for 20 min. The harvested cell pellet was PBS-washed twice (pH 7.4), treated with red blood cell lysis buffer, and the neutrophils were collected by centrifugation at 800 × g for 5 min. Neutrophils were lysed via three alternating −80 °C/25 °C freeze-thaw cycles, followed by 14,000 × g centrifugation to harvest NMs in the pellet, with approximately 3 × 10^6^ NMs yield per 6 ml whole blood.

#### 2.1.3. Fabrication of NM@Ti_3_C_2_T_x_ NPs

For NM@Ti_3_C_2_T_x_ NPs fabrication, the optimized NM@Ti_3_C_2_T_x_ NPs mass ratio (w/w) was 1–1.5:1. In the standard protocol, 20 mg d-Ti_3_C_2_T_x_ was added into 20 mg of 1 mg/ml NMs suspension for complete, efficient membrane coating. The mixture was dispersed and ultrasonicated at 42 kHz for 5 min. Excess unbound NMs were then removed by centrifugation at 10000 × g for 10 min, followed by three washes with Milli-Q water. The collected NM@Ti_3_C_2_T_x_ NPs were lyophilized for storage.

#### 2.1.4. Characterization of NM@Ti_3_C_2_T_x_ NPs

NM@Ti_3_C_2_T_x_ NPs morphology was characterized by transmission electron microscopy (TEM, Talos F20, 80 kV). Dynamic light scattering (DLS, Zetasizer Nano ZS) was used to measure the size distribution and zeta potential. X-ray diffraction (XRD) was performed to verify the crystallinity of d-Ti_3_C_2_T_x_.

#### 2.1.5. In vitro antibacterial assay

The pathogenic *Escherichia coli* (ATCC) was purchased from Beijing Bio-Collection Co., Ltd. *E. coli* cells were grown in LB medium at 37 °C, harvested while in exponential phase, centrifuged (5000 rpm, 10 min, 4 °C), and resuspended in 10 mM phosphate buffer (pH 7.0). *E. coli* (1 × 10^8^ CFU/ml) was treated with PBS, Ti_3_C_2_T_x_, or NM@Ti_3_C_2_T_x_ NPs (0, 20, 50, 100, and 200 μg/ml) for 8 h. Bacterial viability was assessed via colony counting and live/dead staining (SYTO 9/PI).

#### 2.1.6. Cytocompatibility and anti-inflammatory activity

The macrophages (RAW264.7, Rosetta Stone Cell Bank) and human umbilical vein endothelial cells (HUVECs, Keruise Cell Bank) were employed to evaluate the cytotoxicity of NM@Ti_3_C_2_T_x_ NPs. The RAW264.7 cells were grown in Dulbecco's modified Eagle's medium (DMEM) supplemented with 10% fetal bovine serum (FBS), and HUVECs were cultured in ECM complete medium. The cells were treated with NM@Ti_3_C_2_T_x_ NPs (0, 20, 50, 100, and 200 μg/ml) for 24 h, and the viability was evaluated using CCK-8. For anti-inflammatory assays, LPS (200 ng/ml)/IFN-*γ* (2.5 ng/ml) – stimulated macrophages were treated with NM@Ti_3_C_2_T_x_ NPs (100 μg/ml), and intracellular reactive oxygen species (ROS) levels were quantitatively measured using a 2',7'-dichlorodihydrofluorescein diacetate (DCFH-DA) fluorescence assay.

#### 2.1.7. Cecal ligation and puncture (CLP) model

Isoflurane-anesthetized mice underwent midline laparotomy, with the cecum 50% ligated using a 3–0 suture, an 18-G needle through-and-through puncture, and gentle compression to trigger peritoneal fecal leakage. The cecum was repositioned, and the abdominal incision was closed. The sham group received identical anesthesia and laparotomy without ligation or puncture. The cecum was located, but neither ligation nor perforation was performed. The abdominal incision was closed routinely, followed by fluid resuscitation. Post-operatively, mice were kept in thermostatic cages to avoid hypothermia during anesthesia recovery. Fully awake animals were returned to the animal facility for standard care with ad libitum access to food and water. Post-surgery, 28 mice were randomly assigned into 4 groups (*n* = 7): sham, CLP, Ti_3_C_2_T_x_, and NM@Ti_3_C_2_T_x_ NPs. Equal group sizes were guaranteed via computer-generated block randomization (block size = 4), with allocation concealment implemented by an independent researcher who sealed group assignments in sequentially numbered opaque envelopes before intervention to eliminate selection bias. Four mice per group were monitored for 7 days for survival rate and MSS (Shrum et al. 2014) (A scoring system to evaluate sepsis severity in a mouse model comprises seven variables: appearance, level of consciousness, activity, response to stimulus, eyes, respiration rate, and respiration quality.). Each variable is rated on a scale from 0 to 4 based on severity, yielding a total score ranging from 0 to 28, body weight, and body temperature. The remaining three mice in each group were euthanized 24 h post-surgery to collect blood samples, peritoneal lavage fluid, and liver tissue for the analysis of inflammatory markers (TNF-*α*, IL-1β, MDA, IL-6, and IL-10).

#### 2.1.8. In vivo biodistribution and histology

Mice were randomly allocated into 2 groups (*n* = 4): sepsis DiR group (DiR group) and sepsis NM@Ti_3_C_2_T_x_ NPs group (NM@Ti_3_C_2_T_x_ NPs group). DiR-labeled NM@Ti_3_C_2_T_x_ NPs were intravenously injected into CLP mice, and their in vivo biodistribution was tracked via fluorescence imaging (FLM) using an IVIS imaging system. The imaging parameters were set as follows: fluorescence mode, mouse subject height 1.50 cm, DiR as the fluorescent probe, excitation wavelength 748 nm, and emission wavelength 780 nm. Major organs were harvested ex vivo for HE and immunohistochemical staining. The dosage of NM@Ti_3_C_2_T_x_ NPs used across all in vivo experiments was 10 mg/kg, calculated based on Ti_3_C_2_T_x_ mass.

### 2.2. Reagents

All chemicals and reagents were of analytical grade and used as received without further purification. Recombinant mouse M-CSF (315-02-10UG, Thermo Fisher Scientific, USA) and Ti₃AlC₂ powder (910775, Sigma-Aldrich, USA) were obtained from the indicated suppliers. A full panel of assay kits, including MDA lipid peroxidation assay kit (S0131), DAPI nuclear stain (C1002), DCFH-DA ROS probe (S0033S), bacterial live/dead staining kit (C2030S), antifade mounting medium (P0131), QuickBlock™ blocking buffer (P0228), and red blood cell lysis buffer (R1010), was purchased from Beyotime Biotechnology (Jiangsu, China). IL-1β (JM-02323M2) and TNF-*α* (JM-02415M2) ELISA kits were supplied by Jiangsu Jingmei Biotechnology (Jiangsu, China), while IL-6 (SYP-M0031QX) and IL-10 (SYP-M0033) ELISA kits were obtained from UpingBio (Shenzhen, China). The additional reagents included BCA protein assay kit (BL521A, Biosharp, Beijing, China), 4% tissue fixative (P1110, Solarbio Life Sciences, Beijing, China), sodium chloride (Sinopharm Chemical Reagent Co., Ltd., China), lymphocyte separation medium (07851, Stemcell, Canada), PBS (E607008-0500, Sangon Biotech, China), LiF (L812327, Macklin, China), and DiR fluorescent dye (abs45153692, Absin, China). Ultrapure deionized water was used for all experimental procedures.

### 2.3. Statistical analysis

All statistical analyses were performed in GraphPad Prism 9 (GraphPad Software, USA). The normality of all continuous variables was verified via the Shapiro-Wilk test, and all datasets passed normality (all *P* > 0.05). Comparisons between two groups used the independent samples t-test, while comparisons across three or more groups adopted one-way ANOVA with appropriate post-hoc pairwise testing. Normally distributed continuous data are presented as the mean ± standard deviation (mean ± SD). Survival differences were assessed via the Log-rank test with effect sizes calculated using Cliff’s delta. Statistical significance was defined as *P* < 0.05, denoted as *P < 0.05, **P < 0.01, ***P < 0.001. The study flowchart was prepared in Adobe Illustrator (Adobe Systems, USA); the immunofluorescence images were analyzed in ImageJ (NIH, USA); all final figures were assembled using Adobe Photoshop (Adobe Systems, USA).

## 3. Results and discussion

### 3.1. Fabrication and Characterization of NM@Ti_3_C_2_T_x_ NPs

NM@Ti_3_C_2_T_x_ NPs were synthesized via co-centrifugation of delaminated Ti_3_C_2_T_x_ (d-Ti_3_C_2_T_x_) and neutrophil membrane fragments. d-Ti_3_C_2_T_x_ was prepared from Ti_3_AlC_2_ by LiF/HCl chemical etching followed by exfoliation. Neutrophil membranes were extracted from isolated neutrophils via repeated freeze‒thaw cycles and sequential centrifugation. The Tyndall effect confirmed the good colloidal stability and uniform dispersion of d-Ti_3_C_2_T_x_ (Figure 2A). XRD analysis characterized the crystal structure of d-Ti_3_C_2_T_x_. The (002) characteristic peak of Ti_3_C_2_T_x_ appeared at 2θ = 6.05°, downshifted from 9.52° for pristine Ti_3_AlC_2_, and matched JCPDS card No. 52-0875. The red-shifted (002) plane after LiF/HCl etching, together with the complete disappearance of the Ti_3_AlC_2_ (104) peak at approximately 39°, confirmed full Al layer removal and successful exfoliation of Ti_3_C_2_T_x_ (Zhang et al. 2021) (Figure 2B). TEM confirmed that d-Ti_3_C_2_T_x_ presented a typical uniform-thickness, smooth-surface 2D sheet structure. The neutrophil membrane vesicles were spherical, with an average diameter of 110 ± 15 nm (Figure 2D). The resultant neutrophil membrane-coated d-Ti_3_C_2_T_x_ NPs displayed a tight core-shell interfacial binding, verifying stable membrane encapsulation (Figure 2C, 2D, and 2E). Zeta potential analysis further characterized surface charge properties. Ti_3_C_2_T_x_ (hydrodynamic size 164.2 ± 5 nm) had a zeta potential of −34.1 mV, while NM@Ti_3_C_2_T_x_ NPs (220.2 ± 8 nm) showed a more negative value of −41.2 mV, confirming successful membrane coating (Figure 2F). DLS measurements revealed an initial hydrodynamic diameter of the NPs of 152 ± 5 nm with PDI < 0.2 (Figure 2G). After 48 h in PBS, the particle size slightly decreased to 147 ± 8 nm (PDI < 0.25), confirming their stability under physiological conditions (Figure 2H). Then, we evaluated the security of NM@Ti_3_C_2_T_x_ NPs.

The cytotoxicity of NM@Ti_3_C_2_T_x_ NPs was evaluated by co-culturing with RAW264.7 macrophages and HUVECs via CCK-8 assay. After 48 h of incubation, the cell viability remained 85% and 83%, respectively, even at the concentration of 200 μg/ml (Figure 3). These results confirmed the excellent biocompatibility of NM@Ti_3_C_2_T_x_ NPs.

**Figure 2.**
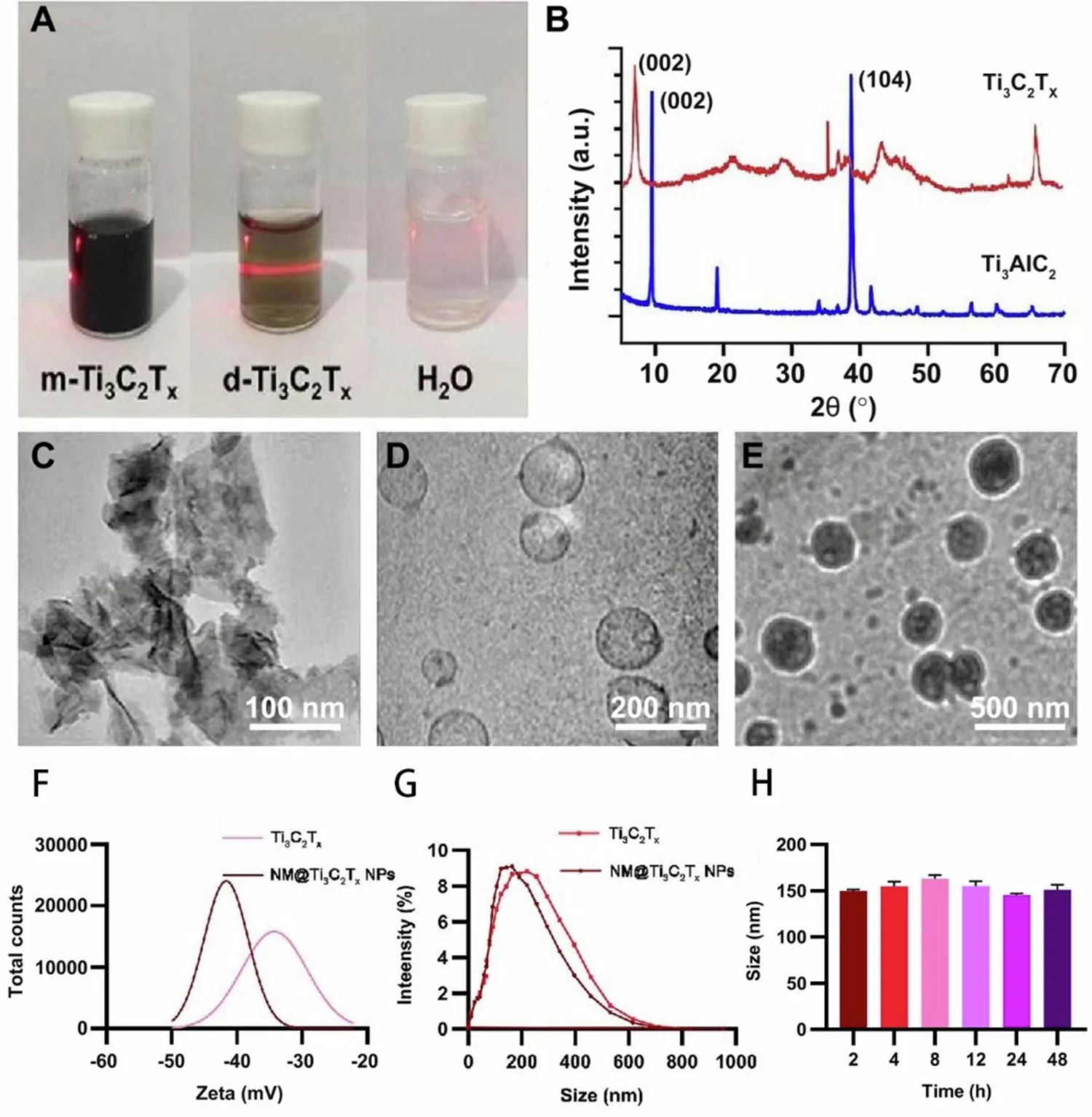
Fabrication and characterization of NM@Ti_3_C_2_T_x_ NPs: (A) Tyndall effect characterization of colloidal d-Ti_3_C_2_T_x_, confirming uniform nanosheet dispersion and satisfactory colloidal stability. (B) XRD pattern of the as-prepared Ti_3_C_2_T_x_. (C) TEM image of d-Ti_3_C_2_T_x_, showing a typical 2D sheet structure with uniform thickness and smooth surface. (D) TEM image of purified neutrophil membrane vesicles. (E) TEM image of NM@Ti_3_C_2_T_x_ NPs, clearly showing a tight core‒shell interface between the neutrophil membrane shell and d-Ti_3_C_2_T_x_ core. (F) Zeta potential measurements of d-Ti_3_C_2_T_x_ and NM@Ti_3_C_2_T_x_ NPs. (G) Comparison of the particle size distributions of d-Ti_3_C_2_T_x_ and NM@Ti_3_C_2_T_x_ NPs. (H) Dynamic particle size stability of NM@Ti_3_C_2_T_x_ NPs in PBS. Data are presented as the mean ± SD (*n* = 3).

**Figure 3.**
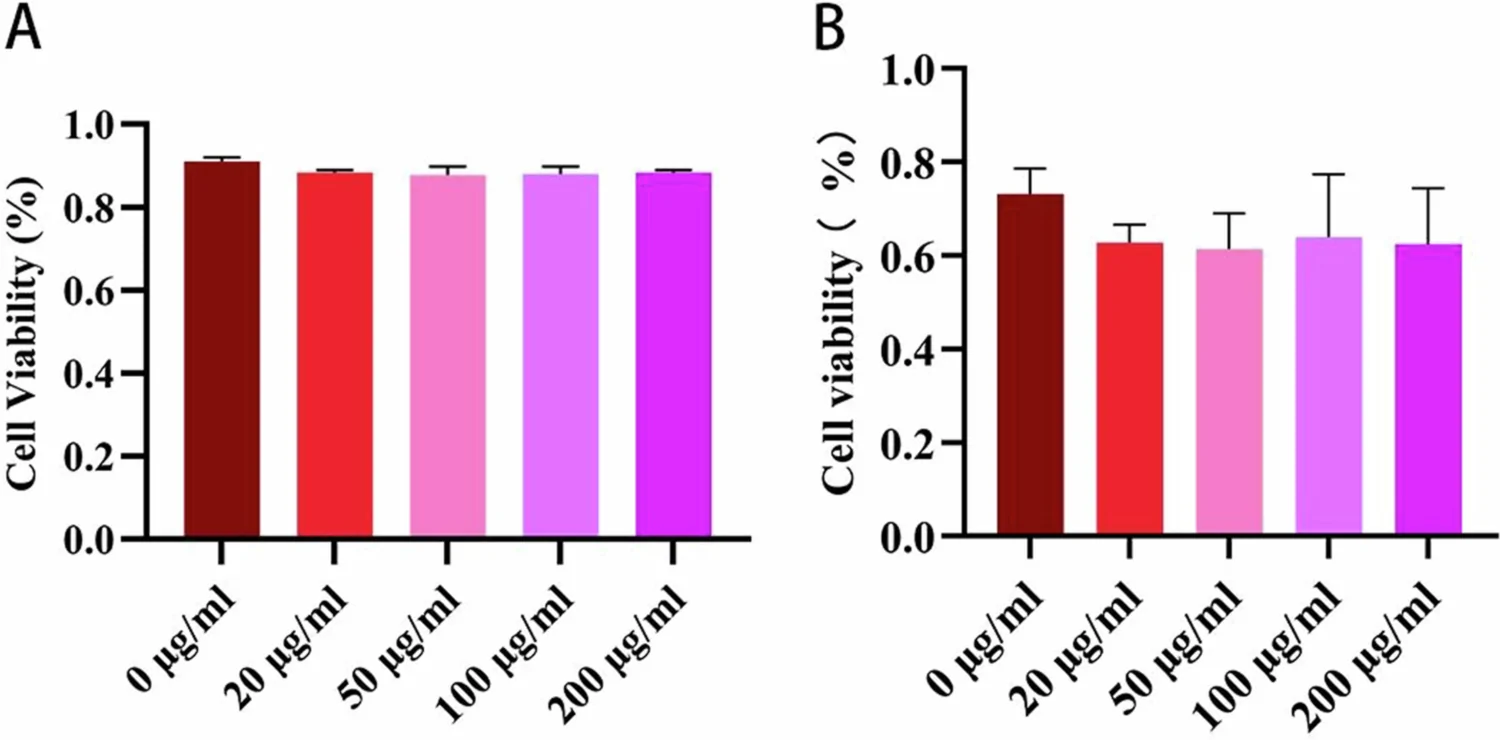
Cell viability. (A) Viability of RAW264.7 macrophages after 24 h of incubation with gradient concentrations of NM@Ti_3_C_2_T_x_ NPs. (B) Viability of HUVECs after 24 h of incubation with gradient concentrations of NM@Ti_3_C_2_T_x_ NPs. Data are presented as the mean ± SD (*n* = 3).

### 3.2. Antibacterial efficacy of NM@Ti_3_C_2_T_x_ NPs in vitro

*E. coli* was selected as the model pathogen to systematically assess the antibacterial performance of NM@Ti_3_C_2_T_x_ NPs. Bacterial colony counting, live/dead staining, and survival rate analysis were performed to verify the antibacterial effects of 0–200 μg/ml (Ti_3_C_2_T_x_ equivalent) NM@Ti_3_C_2_T_x_ NPs after co-incubation (Figure 4).

#### 3.2.1. Bacterial colony count analysis

Untreated *E. coli* formed dense, evenly distributed colonies, indicating robust bacterial proliferation. NM@Ti_3_C_2_T_x_ NPs at 20 μg/ml reduced colony counts by 25.1%, while higher doses (50 and 100 μg/ml) further lowered colony numbers to 37.5% and 16.2% of the PBS control, revealing clear dose-dependent antibacterial activity (Figure 4A and 4C). While Ti_3_C_2_T_x_ at 100 μg/ml reduced colonies by 81.3%, NM@Ti_3_C_2_T_x_ NPs achieved superior efficacy, confirming synergistic antibacterial enhancement from neutrophil membrane coating.

#### 3.2.2. Live/dead bacterial staining

Live/dead staining further validated the antibacterial activity of NM@Ti_3_C_2_T_x_ NPs. Dual staining with SYTO 9 (green, live bacteria) and propidium iodide (PI, red, dead bacteria) clearly visualized its bactericidal effect (Figure 4B). The PBS-treated control groups showed dominant green fluorescence, while red signal intensity in the NM@Ti_3_C_2_T_x_ NPs-treated groups increased in a concentration-dependent manner. After 8 h incubation with 100 μg/ml NM@Ti_3_C_2_T_x_ NPs, quantitative fluorescence analysis showed the proportion of viable bacteria dropped below 25% (Figure 4D).

**Figure 4.**
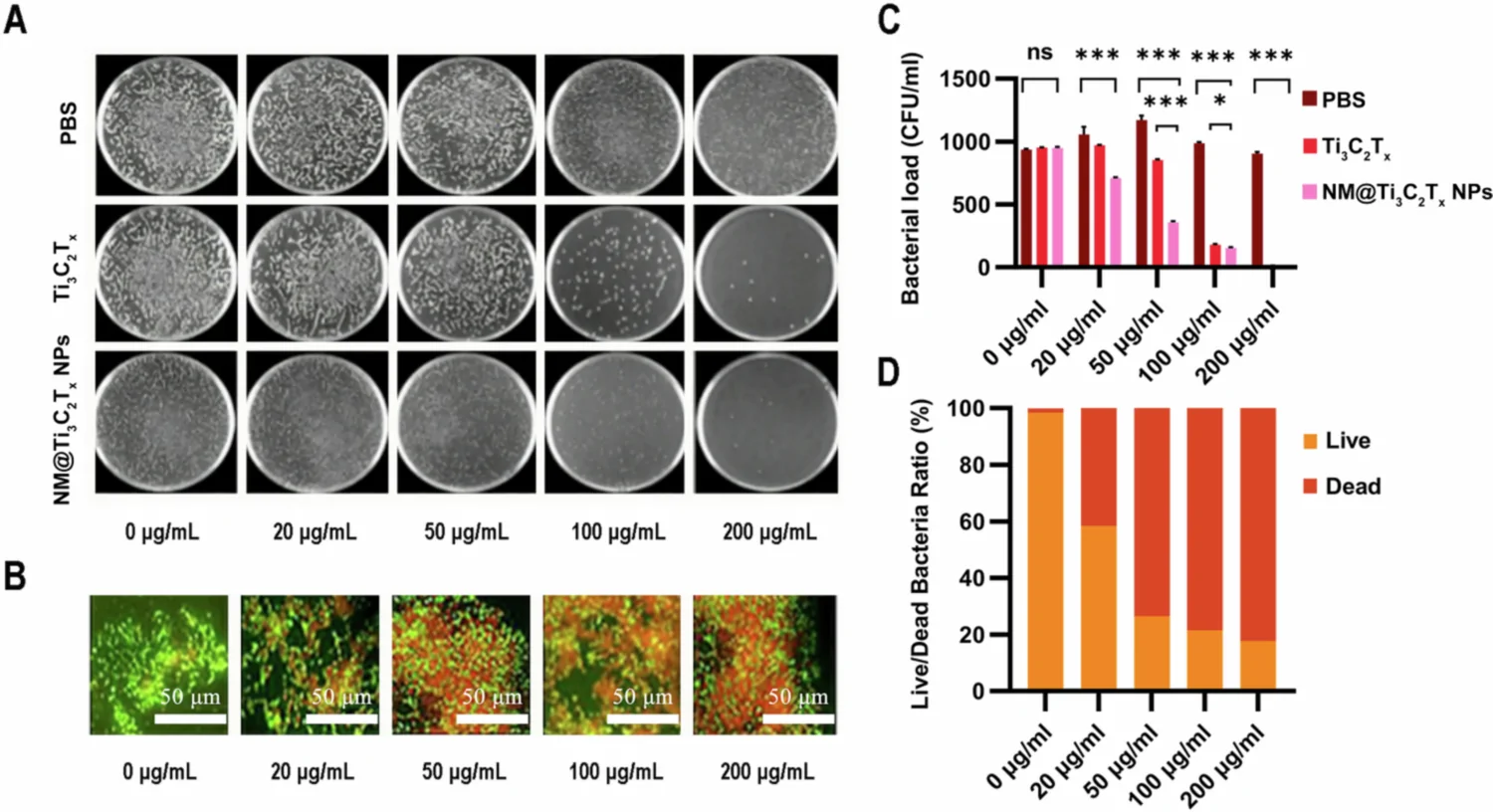
Antibacterial assays: (A) colony counting images of *E. coli* after 24 h treatment with NM@Ti_3_C_2_T_x_ NPs at 0, 20, 50, 100, and 200 μg/ml. (B) Live/dead immunofluorescence staining images of *E. coli* after 8 h treatment with NM@Ti_3_C_2_T_x_ NPs at 0, 20, 50, 100, and 200 μg/ml. (C) Statistical analysis of *E. coli* colony counts for PBS, NM@Ti_3_C_2_T_x_ NPs, and Ti_3_C_2_T_x_ groups after 24 h treatment. NM@Ti_3_C_2_T_x_ NPs exhibited obvious dose-dependent antibacterial activity, with significantly higher bactericidal efficacy than Ti₃C₂T_x_ at the same 100 μg/mL concentration (*n* = 3, *P < 0.05, ***P < 0.001, and ns *P* > 0.05). (D) Quantitative live/dead bacterial ratio analysis based on the corresponding fluorescence images. Data are presented as the mean ± SD (*n* = 3).

### 3.3. Antioxidant effects of NM@Ti_3_C_2_T_x_ NPs in vitro

DCFH-DA fluorescence assays showed that LPS-induced ROS levels (358.3 ± 12.7 AU) were reduced by 67.5% to 116.3 ± 16.2 AU after NM@Ti_3_C_2_T_x_ NPs treatment, which was significantly lower than the LPS group (*P* < 0.001) and comparable to Ti_3_C_2_T_x_ (126 ± 27 AU, 64.8% reduction). This result confirms the potent antioxidant capacity of NM@Ti_3_C_2_T_x_ NPs (Figure 5A and 5B).

**Figure 5.**
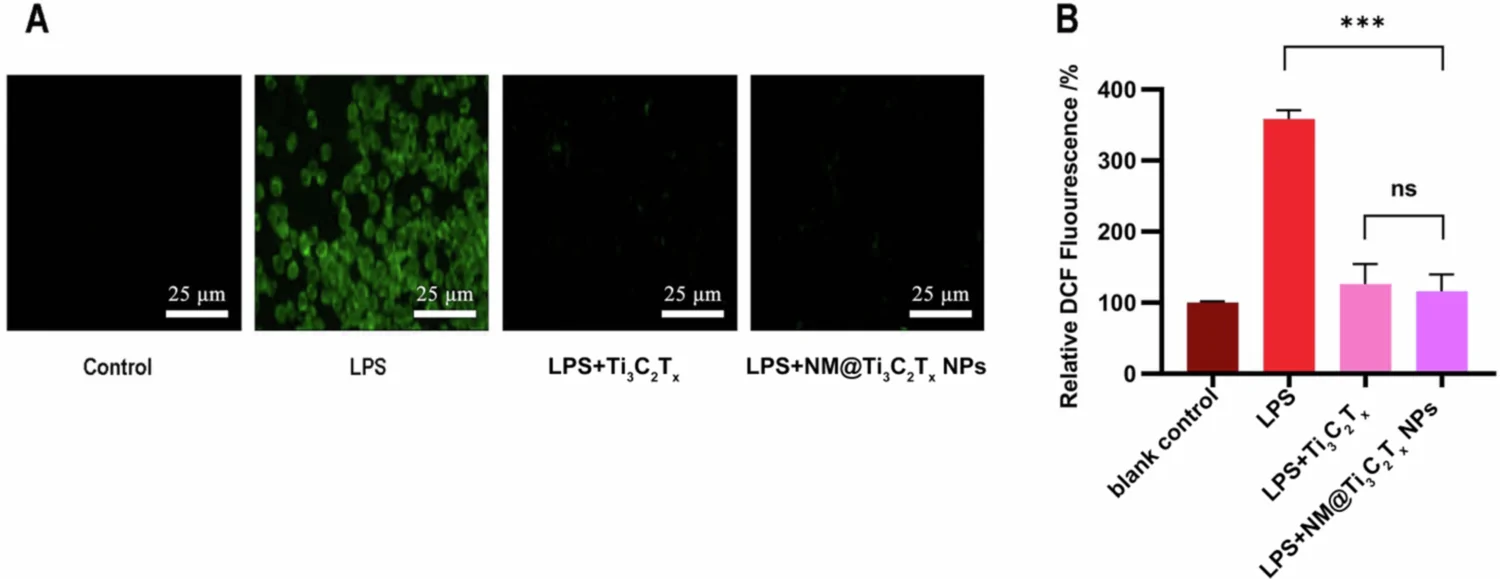
(A) RAW264.7 cells were treated with different drugs and stained with intracellular ROS detected by DCFH-DA. (B) Quantitative analysis of intracellular ROS immunofluorescence staining detected by DCFH-DA (*n* = 3, ***P < 0.001 and ns *P* > 0.05).

### 3.4. Therapeutic efficacy of NM@Ti_3_C_2_T_x_ NPs in a CLP-induced sepsis model

#### 3.4.1. Biodistribution of NM@Ti_3_C_2_T_x_ NPs in CLP-induced sepsis mice in vitro

To investigate the in vivo biodistribution of NM@Ti_3_C_2_T_x_ NPs, CLP model mice received an intraperitoneal injection of DiR-labeled NPs for fluorescence imaging. At 24 h post-injection, intense fluorescence signals were detected in the inflammatory site, confirming rapid targeted accumulation of NM@Ti_3_C_2_T_x_ NPs at the infection locus (Figure 6A). In contrast, free DiR exhibited diffuse fluorescence signals throughout the abdominal cavity shortly after injection, followed by rapid entry into systemic circulation and eventual hepatic metabolism (Figure 6A). Ex vivo imaging of major organs (heart, liver, spleen, lung, kidney, and colon) at 24 h post-injection further confirmed that NM@Ti_3_C_2_T_x_ NPs preferentially accumulated in the cecum and adjacent inflamed tissues, with significantly higher fluorescence intensity than in non-target organs. Detectable signals were still observed in organs such as the liver, indicating partial NP entry into the systemic circulation and unavoidable off-target distribution (Figure 6B). Collectively, these results confirm that NM@Ti_3_C_2_T_x_ NPs preferentially accumulate at inflammatory sites after intraperitoneal administration in CLP models, laying a solid foundation for their subsequent local therapeutic effects.

**Figure 6.**
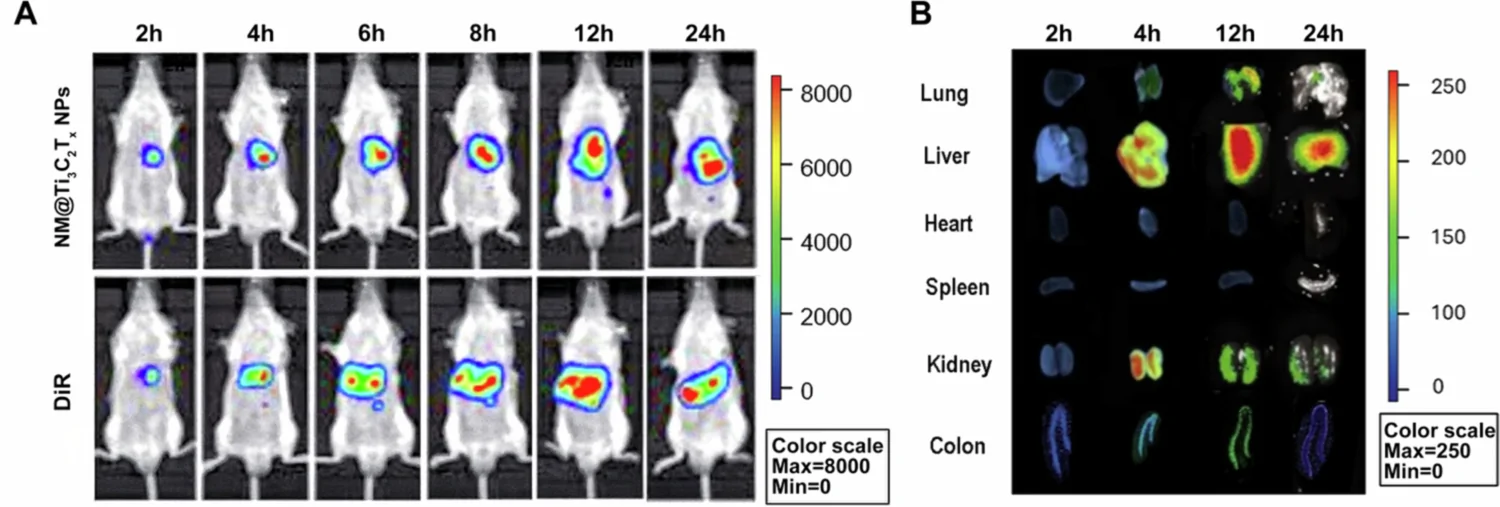
In vivo biodistribution of NM@Ti_3_C_2_T_x_ NPs in CLP-induced septic mice: (A) Real-time fluorescence imaging of mice intravenously injected with NM@Ti_3_C_2_T_x_ NPs or free DiR. (B) Ex vivo fluorescence imaging of major organs (heart, liver, spleen, lung, kidney, and colon) at 24 h post-injection (*n* = 3).

#### 3.4.2. Survival and clinical parameters

The therapeutic efficacy of NM@Ti_3_C_2_T_x_ NPs against sepsis was evaluated in the CLP model. The survival rate, MSS score, body weight, and temperature changes were systematically compared (Figure 7). The CLP model was validated as successfully established, with 72-h survival rates of 66.7% and 33.3% mortality within 24 h. Ti_3_C_2_T_x_-treated septic mice showed 66.7% 72-h survival after surgery, reflecting its limited survival-improving effect. NM@Ti_3_C_2_T_x_ NPs treatment further elevated the 72-h survival rate to 83.3%, demonstrating robust therapeutic potency (Figure 7A).

The MSS score was applied to quantify clinical symptom severity. In the CLP model group, the MSS reached 17.00 ± 12.76 at 24 h post-surgery, indicating the onset of severe sepsis. A total of 66.7% of mice in the Ti_3_C_2_T_x_ group died within 24 h after their MSS elevated to 28, while NM@Ti_3_C_2_T_x_ NPs treatment reduced the MSS to 12.67 ± 13.32, confirming effective alleviation of sepsis symptoms (Figure 7B).

After successful CLP modeling, mice housed at 20–26 °C showed an initial transient temperature drop, followed by a rapid febrile response, and ultimately progressed to fatal hypothermia due to impaired thermoregulation. In the CLP group, body weight decreased to 14.50 ± 1.32 g, and core temperature increased to 38.03 ± 0.31 °C (approximately 4.68%) at 12 h post-surgery, confirming metabolic disturbance and thermoregulatory dysfunction. In the Ti_3_C_2_T_x_ group, body weight was reduced by 20.83 ± 8.40% to 15.63 ± 1.68 g, with a mild temperature rise to 37.73 ± 0.61 °C at 12 h. NM@Ti_3_C_2_T_x_ NPs-treatment maintained core temperature at 37.76 ± 0.25 °C without hypothermia, indicating significantly restored metabolic stability and thermoregulatory function (Figure 7C and 7D).

**Figure 7.**
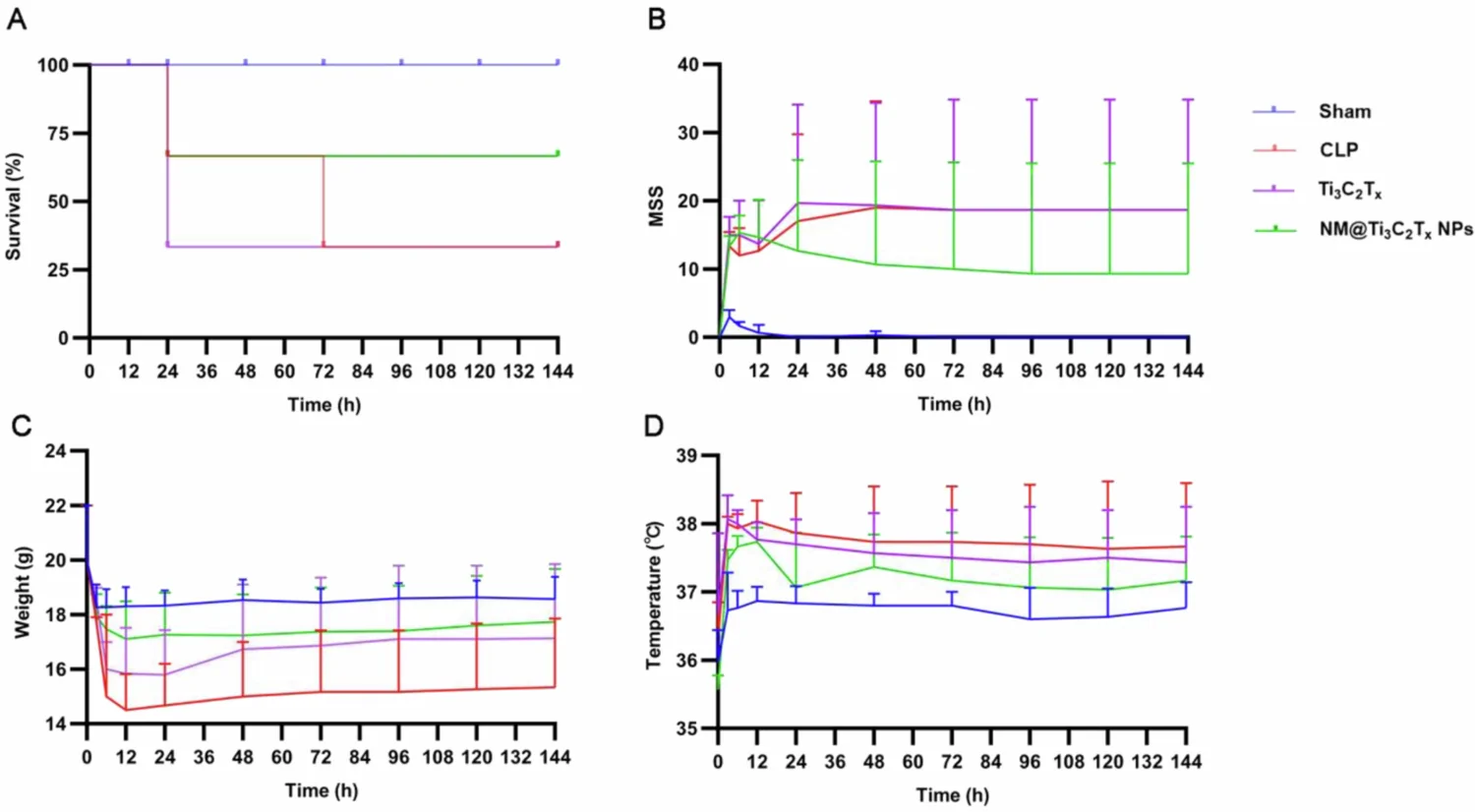
(A) 72 h survival curve. (B) MSS scoring. (C) Body weight dynamics. (D) Core body temperature variation. Data were expressed as the mean ± SD (*n* = 3).

#### 3.4.3. The regulatory effects of NM@Ti_3_C_2_T_x_ NPs on bacterial load, oxidative stress, and inflammatory factors in septic mice

To further elucidate the therapeutic mechanism of NM@Ti_3_C_2_T_x_ NPs in sepsis, bacterial load, oxidative stress marker MDA, and inflammatory factors IL-1β and TNF-*α* in mice were analyzed at 24 h post-modeling (Figure 8). The bacterial load in the peritoneal lavage fluid was quantified via a plate colony counting assay for all groups (Figure 8A). The results showed that the CLP group had a significantly higher bacterial load than the sham group, at 2.73 ± 0.25 × 10^5^ CFU/ml vs. 1.10 ± 0.45 × 10^5^ CFU/ml, confirming successful induction of bacterial infection in the CLP model. Ti_3_C_2_T_x_ treatment reduced bacterial load by 15.7% to 2.30 ± 0.70 × 10^5^ CFU/ml compared with the CLP group, while NM@Ti_3_C_2_T_x_ NPs treatment further lowered it to 2.03 ± 0.62 × 10^5^ CFU/ml (25.6% reduction, 11.7% lower than Ti_3_C_2_T_x_), indicating the neutrophil membrane coating enhanced the targeting specificity of Ti_3_C_2_T_x_ to boost its antibacterial potency.

Oxidative stress was analyzed by detecting serum malondialdehyde (MDA) levels (Figure 8B). The CLP group showed a significantly elevated MDA level of 7.83 ± 0.23 nmol/ml, versus 2.00 ± 0.10 nmol/ml in the sham group, confirming severe sepsis-induced oxidative damage. After NM@Ti_3_C_2_T_x_ NPs treatment, the MDA level significantly decreased to 3.08 ± 0.12 nmol/ml (a decrease of 60.7%, *P* < 0.0001), which was significantly better than the Ti_3_C_2_T_x_ group (5.55 ± 0.20 nmol/ml, *P* < 0.0001), suggesting that NM@Ti_3_C_2_T_x_ NPs effectively alleviated sepsis-related oxidative stress damage through a synergistic antioxidant mechanism. The analysis of inflammatory factors was performed. The levels of inflammatory factors in the serum and liver tissue homogenate were detected by ELISA. CLP challenge significantly elevated the serum levels of pro-inflammatory cytokines IL-1β, TNF-*α*, and IL-6 compared with those in the sham group. Relative to the Ti_3_C_2_T_x_ group, NM@Ti_3_C_2_T_x_ NPs further reduced serum IL-1β by 51.4%, TNF-*α* by 56.3%, and IL-6 by 38.4%, with identical downward inhibitory trends consistently reproduced in liver tissue homogenates: NM@Ti_3_C_2_T_x_ NPs downregulated hepatic IL-6 by 71.9%, showing statistically superior anti-inflammatory efficacy to Ti_3_C_2_T_x_ across all pro-inflammatory markers (Figure 8C, 8D, 8E, 8F, 8G, and 8H). This unified observation confirms that the neutrophil membrane coating markedly amplifies the nanoparticles’ ability to suppress excessive systemic pro-inflammatory cascades. To further characterize the immunomodulatory profile of NM@Ti_3_C_2_T_x_ NPs, the serum and liver anti-inflammatory cytokine IL-10 was quantified. CLP induced dramatic IL-10 elevations (*P* < 0.0001), confirming overactivated the compensatory anti-inflammatory response in the septic model. NM@Ti_3_C_2_T_x_ NPs intervention substantially reversed this pathological overactivation, reducing the IL-10 levels in the circulation and liver to significantly lower values than those in the CLP and Ti_3_C_2_T_x_ groups (Figure 8I and Figure 8J). Consistent with pro-inflammatory cytokine profiles, these multi-cytokine data verify that NM@Ti_3_C_2_T_x_ NPs restore sepsis-associated immune homeostasis via concurrent suppression of overactivated anti-inflammatory signaling and excessive pro-inflammatory mediator release.

**Figure 8.**
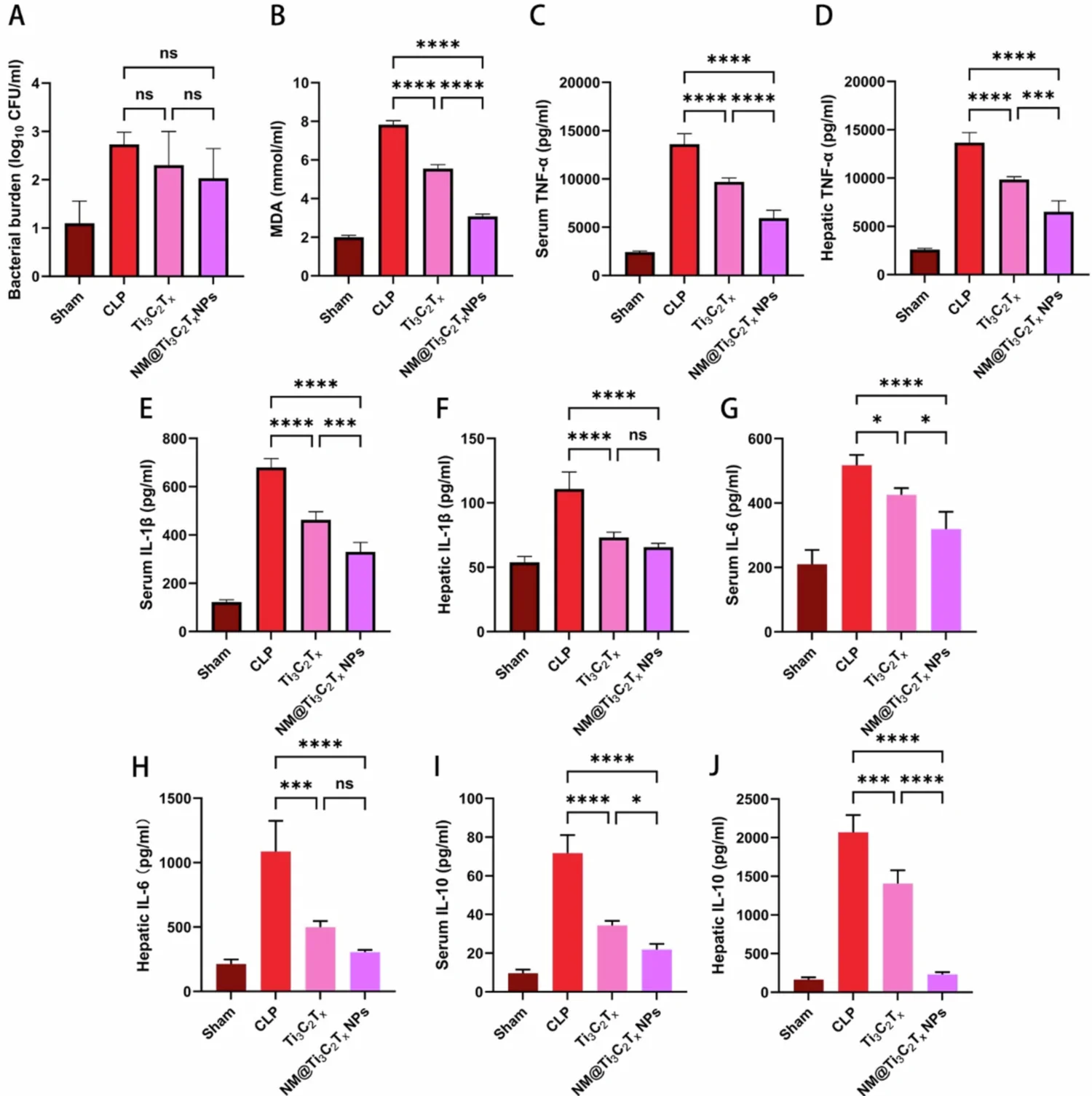
Changes in bacterial load and different groups (the sham group, the CLP group, the Ti_3_C_2_T_x_ group, and the NM@Ti_3_C_2_T_x_ NPs group) of mice. (A) Bacterial load in the peritoneal lavage fluid. (B) MDA content in the serum. (C) Serum TNF-*α* level. (D) Hepatic TNF-*α* level. (E) Serum IL-1β level. (F) Hepatic IL-1β level. (G) Serum IL-6 level. (H) Hepatic IL-6 level. (I) Serum IL-10 level. (J) Hepatic IL-10 level. Data were expressed as the mean ± SD (*n* = 4, *P < 0.05, ***P < 0.001, ****P < 0.0001 and ns *P* > 0.05).

#### 3.4.4. Histopathological changes in septic mice treated with NM@Ti_3_C_2_T_x_ NPs

To systematically evaluate the organ-protective effect of NM@Ti_3_C_2_T_x_ NPs in septic mice, HE staining was performed on lung, liver, kidney, and colon tissues from all groups for histopathological examination (Figure 9).

The sham group showed intact tissue and cellular architecture with negligible pathological changes and minimal inflammatory infiltration. The CLP group presented severe structural disruption and extensive inflammatory cell infiltration, confirming acute organ injury. The Ti_3_C_2_T_x_ group showed partial amelioration of tissue damage and reduced inflammatory infiltration. The NM@Ti_3_C_2_T_x_ NPs group exhibited largely restored tissue architecture, with only mild damage and markedly decreased inflammatory infiltration, representing notable pathological improvement, among which lung tissue showed the least pathological damage across all treatment groups.

**Figure 9.**
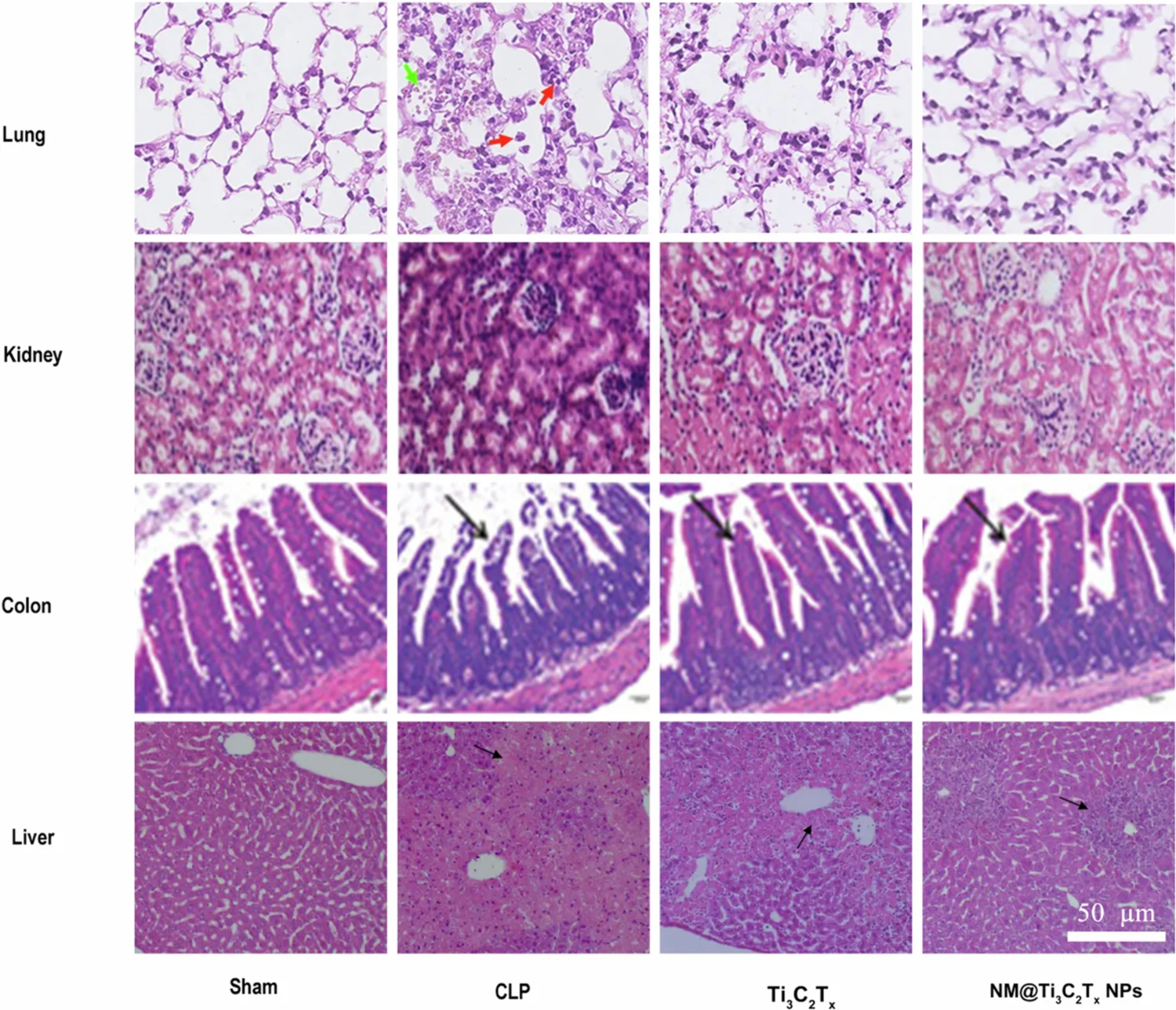
Histopathological evaluation of NM@Ti₃C₂Tx NPs against CLP-induced multiple organ injury in septic mice. HE staining was performed on lung, liver, kidney, and colon tissues collected at the experimental endpoint for light microscopic observation of pathological alterations. Representative images show that the sham group had intact tissue architecture with negligible inflammatory infiltration. The CLP group developed severe structural disruption and extensive inflammation. Ti_3_C_2_T_x_ treatment moderately ameliorated pathological abnormalities, while NM@Ti_3_C_2_T_x_ NPs treatment largely restored the tissue structure with only mild damage and markedly reduced inflammation, with the most prominent improvement observed in the lung tissue. (*n* = 4, scale bar: 50 μm).

## 4. Discussion

Sepsis is an infection-induced systemic inflammatory response syndrome that rapidly progresses to high-mortality multiple organ dysfunction (Rudd et al. 2020; Liu et al. 2022). Current clinical regimens, predominantly antibiotics plus symptomatic support, face three core bottlenecks: rising bacterial resistance, incomplete inflammatory storm blockade, and absence of targeted interventions. Developing novel high-efficiency sepsis therapeutics thus holds great clinical value. Here, we constructed NM@Ti_3_C_2_T_x_ NPs, validated their translational potential via systematic characterization, in vitro functional assays, and in vivo efficacy assessment in a septic mouse model, and contextualize these findings with the existing literature below.

### 4.1. Interpretation of core research findings

Few-layer delaminated d-Ti_3_C_2_T_x_ was successfully prepared via LiF/HCl etching and exfoliation. XRD confirmed the base material synthesis: the characteristic Al-layer peak of pristine Ti_3_AlC_2_ completely disappeared, and the (002) peak downshifted, matching reported etched Ti_3_C_2_T_x_ MXene signatures. Neutrophil membrane coating was achieved by co-centrifugation, with multiple characterizations verifying successful NM@Ti_3_C_2_T_x_ NPs fabrication. TEM revealed a distinct core-shell structure with membranes tightly wrapping d-Ti_3_C_2_T_x_. Post-coating, zeta potential dropped from −34.1 to −41.2 mV, and the hydrodynamic size increased from 164.2 to 220.2 nm, consistent with typical membrane-coated nanoparticle behavior. Stability assays confirmed NM@Ti_3_C_2_T_x_ NPs retained excellent monodispersity and colloidal stability after 48 h storage in PBS. Cytotoxicity assays confirmed >85% RAW264.7 macrophage viability and >83% HEVUC viability even at 200 μg/ml, verifying the NPs meet in vivo biocompatibility requirements. Compared with Ti_3_C_2_T_x_, neutrophil membrane coating attenuates non-specific in vivo interactions, improves colloidal stability and biosafety, and enables subsequent in vivo application. In vitro assays validated NM@Ti_3_C_2_T_x_ NPs show superior dose-dependent antibacterial activity while fully preserving Ti_3_C_2_T_x_’s intrinsic high antioxidant capacity. At 100 μg/ml, NM@Ti_3_C_2_T_x_ NPs reduced *E. coli* survival to <25% and eliminated 67.5% of LPS-induced ROS, both outperforming equivalent-concentration d-Ti_3_C_2_T_x_.

NM@Ti_3_C_2_T_x_ NPs markedly improved septic mouse outcomes: stabilized body temperature and weight, reduced clinical sepsis scores, and elevated 72 h survival. Mechanistically, they reduced the peritoneal bacterial load by 25.6% and the serum MDA by 60.7%. NM@Ti_3_C_2_T_x_ NPs significantly downregulated pro-inflammatory cytokines (IL-1β, TNF-*α*, and IL-6) in CLP mouse serum and liver, while blunting sepsis-induced compensatory IL-10 elevation. This cytokine profile confirms that NM@Ti_3_C_2_T_x_ NPs restore sepsis immune homeostasis by suppressing excessive pro-inflammatory mediators and overactivated anti-inflammatory signaling. Ultimately, this treatment alleviated pathological damage in the lung and liver, with the most prominent improvement in lung tissue.

This strategy aligns with the current multi-target synergistic paradigm for sepsis treatment, overcoming the core limitation of single antibiotics: bactericidal action without inflammation resolution or inflammatory storm blockade. Compared with Ti_3_C_2_T_x_, NM@Ti_3_C_2_T_x_ NPs outperform across all key endpoints, fully validating the translational value of this biomimetic modification.

### 4.2. Innovative value of this study

Ti_3_C_2_T_x_ MXene nanomaterials have drawn extensive biomedical interest for their large specific surface area, tunable surface chemistry, and intrinsic antioxidant and antibacterial activities. Biomimetic cell membrane modification is an emerging nanoformulation strategy. Neutrophil membranes possess natural inflammatory chemotaxis and have been deployed to treat chronic inflammatory conditions, including rheumatoid arthritis and atherosclerosis, yet NM@Ti_3_C_2_T_x_ NPs have not been explored for sepsis therapy to date.

This work first integrates neutrophil membrane inflammatory tropism with Ti_3_C_2_T_x_’s inherent antibacterial and antioxidant properties, systematically validating NM@Ti_3_C_2_T_x_ NPs’ efficacy and safety for sepsis therapy. This study expands MXene’s biomedical application scope, provides new insights for sepsis nanomedicine, and delivers high basic research value and strong clinical translational potential. Compared with reported sepsis nanotherapies, this design has unique advantages: conventional antioxidant nanoparticles (e.g. CeO₂ NPs, MOF-derived platforms) act solely via ROS neutralization (Chen et al. 2025), while NM@Ti_3_C_2_T_x_ NPs achieve superior multi-organ protection by coupling potent intrinsic antioxidation with inflammatory tropism to resolve the macrophage-driven inflammatory imbalance. LPS-scavenging nanomaterials (e.g. polycationic adsorbents) typically show high off-target risks and systemic side effects (Chen et al. 2025), while NM@Ti_3_C_2_T_x_ NPs specifically accumulate at inflammatory sites relying on the natural inflammatory targeting of neutrophil membranes to regulate innate immune cell activation, reducing the risk of systemic adverse reactions; antibiotic or anti-inflammatory drug-loaded nano-delivery systems rely on exogenous drug loading and carry potential risk of bacterial resistance after long-term application (Elbehiry and Abalkhail 2025), whereas this formulation delivers intrinsic dual antibacterial and anti-inflammatory activities without extra antibiotic loading, mechanistically circumventing resistance.

### 4.3. Limitations of the study and future prospects

This study has several limitations to be addressed in future work. The current CLP sepsis in vivo study lacks an independent antibiotic control group, partially restricting a full evaluation of the regimen’s clinical relevance (Poellmann et al. 2018a). Follow-up work will add this control, follow standardized CLP protocols, and perform systematic immune assessment to further define the formulation’s efficacy profile and safety boundaries. The mouse CLP model, though the most widely used preclinical sepsis platform, it cannot fully recapitulate the complex, comorbid etiology of human clinical sepsis, and the current conclusions require further validation in large animal models (swine, non-human primates) and subsequent clinical trials to confirm human efficacy and safety. Long-term safety assessment of NM@Ti_3_C_2_T_x_ NPs is also incomplete: only short-term efficacy and acute toxicity were evaluated, with no systematic characterization of long-term in vivo metabolism and potential chronic toxicity from organ retention, so Ti_3_C_2_T_x_’s in vivo clearance pathway and long-term safety remain unclarified. Ti_3_C_2_T_x_’s antioxidant activity has been cross-validated in prior studies via electron paramagnetic resonance (EPR) and oxidative stress marker quantification, consistent with our DCFH-DA results, and follow-up work will add dedicated NP-DCF probe co-incubation interference assays to confirm this conclusion (Geng et al. 2022; Yang et al. 2024; Wang et al. 2026). Last but not least, the translational potential of the current preparation process needs to be improved: this study adopted a small-scale co-centrifugation method for laboratory preparation, and the batch stability and cost control of large-scale production still need further optimization to lay a foundation for subsequent translational applications.

## 5. Conclusion

This work develops NM@Ti_3_C_2_T_x_ NPs system integrating active targeting, physical antibacterial, chemical anti-inflammatory, and immunomodulatory functions, representing a novel multi-mechanism sepsis therapeutic strategy. *E. coli* co-culture confirms its concentration-dependent superior bactericidal performance; gradient concentration incubation for 48 h shows no significant viability drop in RAW264.7 macrophages and HEVUCs, verifying favorable biocompatibility. In vitro anti-inflammatory assays validate its enhanced inflammatory factor clearance and superior anti-inflammatory capacity. In CLP model mice, NM@Ti_3_C_2_T_x_ NPs markedly improve survival and mitigate multi-organ damage, with particularly pronounced lung tissue repair. In conclusion, NM@Ti_3_C_2_T_x_ NPs are a promising novel sepsis therapeutic based on a coordinated multi-mechanism biomimetic design. Further safety optimization, in-depth mechanistic validation, and translational adaptability assessment will accelerate its bench-to-bedside progress.

## Supplementary Material

Supplementary Material ARRIVE.pdf

Supplementary Material Revision unmarked.docx

## Data Availability

The data supporting the findings of this study will be made available by the corresponding author upon reasonable request.
